# Scrub Typhus Presenting as Bilateral Sixth Nerve Palsy

**DOI:** 10.7759/cureus.56692

**Published:** 2024-03-22

**Authors:** Sukriti Bhattacharjee, Bibhu Debbarma, Rajesh K Debbarma, Gourab Das

**Affiliations:** 1 Internal Medicine, Agartala Government Medical College, Agartala, IND

**Keywords:** scrub infection in india, no eschar in scrub, scrub typhus case report, sixth cranial nerve palsy, bilateral sixth nerve palsy

## Abstract

Scrub typhus, a tropical rickettsial infection, can have various neurological manifestations. Here, we present the case of an otherwise healthy 19-year-old female who presented to the emergency department with fever, headache, and diplopia. On examination, she was found to have bilateral sixth nerve palsy and bilateral papilledema. Initial unenhanced CT of the brain and CT venogram were unremarkable. The cerebrospinal fluid study was normal. Later, bloodwork revealed high titers of *Orientia tsutsugamushi* antibody. A thorough physical examination revealed no evidence of any eschar. She was treated with doxycycline and had significant clinical improvement with partial recovery of bilateral lateral rectus function. We want to highlight the importance of maintaining a high index of suspicion for atypical neurological presentations of scrub typhus.

## Introduction

Scrub typhus is a mite-born rickettsial zoonotic disease caused by *Orientia tsutsugamushi*. This acute febrile illness can have various neurological manifestations, including meningitis, encephalitis, transverse myelitis, and, rarely, cranial nerve palsies. Involvement of bilateral abducens nerves is quite rare. Due to its variable presentation, the diagnosis and treatment of scrub typhus is usually delayed. Hence, scrub typhus should always be considered in any case of acute undifferentiated fever, including those presenting with focal neurological deficits, especially in India, which is a part of the “Tsutsugamushi triangle.” Here, we report the case of a 19-year-old female who presented with fever, headache, diplopia, and bilateral abducens nerve palsy along with bilateral papilledema. After extensive investigations and neuroimaging, she was finally diagnosed with scrub typhus associated with bilateral abducens nerve palsy, which responded to doxycycline therapy.

## Case presentation

A 19-year-old female from rural northeast India presented to the emergency department with a history of fever, headache, nausea, and vomiting for five days, which was then followed by sudden-onset diplopia for two days. Her past medical history was unremarkable and there was no recent history of travel. On examination, she was febrile and mildly dehydrated but was otherwise conscious and well-oriented to time, place, and person. Further neurological examination revealed a bilateral lateral rectus palsy. On the fundus examination, bilateral papilledema was seen. Kernig’s and Brudzinski’s signs were absent. The rest of the central nervous system and systemic examination revealed no other significant abnormalities.

Blood investigations showed normal hemoglobin, hematocrit, platelets, and a normal total leucocyte count. Liver function tests and kidney function tests were also within normal limits. Chest X-ray was normal. Routine examination of urine was insignificant. Blood and urine cultures also came out to be negative. Initial non-contrast-enhanced CT imaging of the brain was normal. This was followed by a non-contrast MRI of the brain and orbits and a CT venogram, both of which were normal. Contrast MRI could not be performed due to financial and resource constraints.

**Figure 1:**
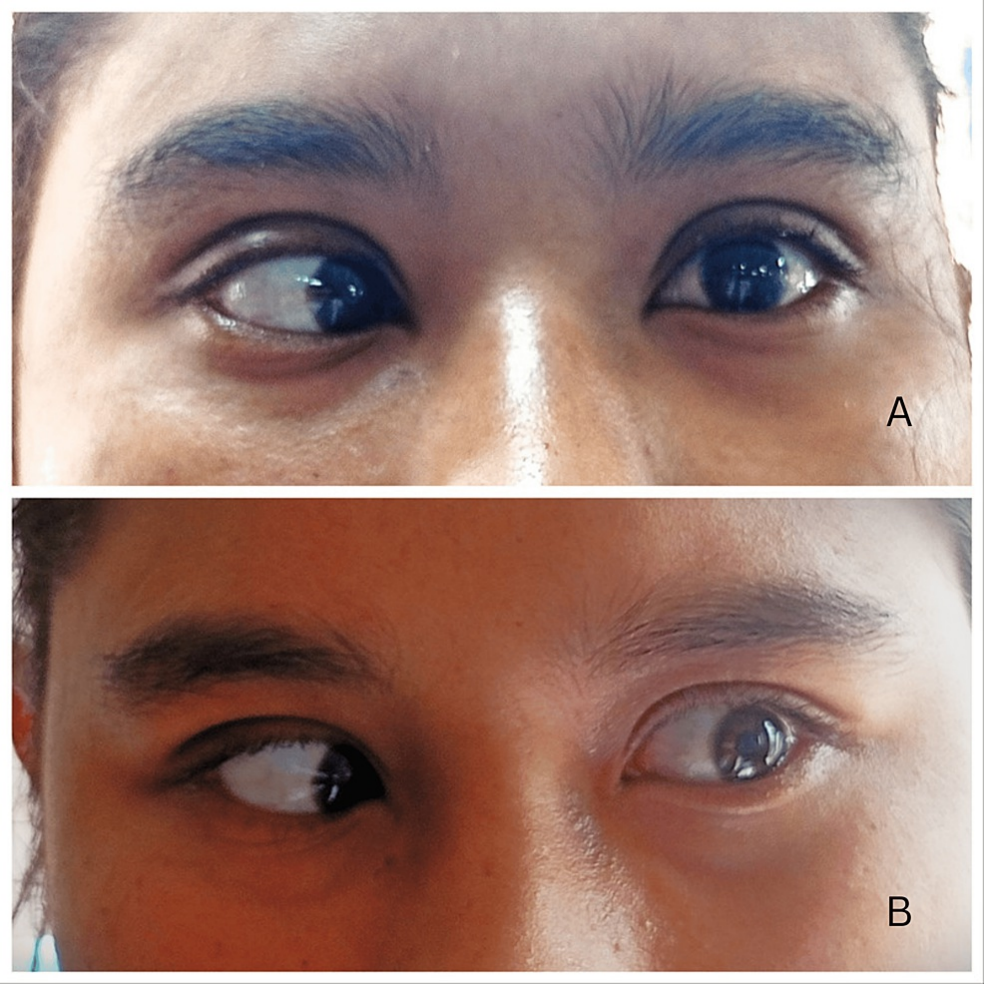
(A) Left lateral gaze of the patient at presentation with sixth nerve palsy. (B) Significant improvement at discharge.

A lumbar puncture revealed clear cerebrospinal fluid which on analysis showed normal cell count, normal protein, and glucose. Cerebrospinal fluid (CSF) opening pressure could not be measured due to resource constraints. CSF culture came out to be negative. Peripheral blood smear showed no malarial parasite. IgM enzyme-linked immunosorbent assay (ELISA) for dengue and Japanese encephalitis was negative. IgM ELISA for scrub typhus was found to be positive. Following this, a thorough physical examination was performed but no eschar was found. The patient was started on a course of 100 mg of intravenous doxycycline twice a day, following which her fever subsided in three days with gradual alleviation of double vision. She partially regained power in both her lateral rectus muscles (Figure 1) over the course of 10 days of hospitalization and was discharged with advice to follow up in the outpatient department.

## Discussion

Scrub typhus, caused by the rickettsial bacterium *Orientia tsutsugamushi* commonly found in the Tsutsugamushi triangle, is a serious public health problem. It has an incidence of 4.6/100,000/10 years with a case fatality risk of up to 13.6% [1]. Bite of the larvae of trombiculid mites (chiggers) transmits the causative gram-negative bacteria which causes disseminated vasculitis and perivascular inflammation ultimately leading to significant vascular leakage and end-organ injury. After an incubation period of 6-21 days, the clinical features manifest as fever, headache, myalgia, and gastrointestinal symptoms. An eschar may be present which usually begins as a primary papule that later crusts to form a black ulcer with central necrosis [2].

Non-specific symptoms in the form of headaches, fever, and myalgia are the most common features. Infected individuals may also develop generalized lymphadenopathy and gastrointestinal symptoms. Systemic manifestations of the disease may start to develop toward the beginning of the second week ranging from central nervous system manifestations, such as acute diffuse encephalomyelitis, encephalopathy, meningitis, and sometimes hearing loss, cranial nerve palsies, and several ocular manifestations [3-5]. Isolated cranial nerve involvement in scrub typhus is rare and thought to be caused by micro-infarction of nerves due to scrub typhus-induced vasculitis in vasa vasorum as well as stretching of the vessels due to dilated ventricles [6-8]. Involvement of abducens nerve in scrub typhus is quite uncommon. Rickettsial fever presenting with isolated third nerve palsy has been reported [9]. Acute sensorineural hearing loss and severe otalgia due to eighth nerve involvement in scrub typhus have also been described [10]. Indirect fluorescent antibody testing remains the gold standard for serological diagnosis of scrub typhus, although the preferred test is ELISA owing to its high specificity and sensitivity [11].

In our patient diagnosed with scrub typhus along with bilateral sixth nerve palsy, doxycycline therapy led to significant recovery of bilateral lateral rectus palsy and alleviation of symptoms.

## Conclusions

Scrub typhus, a common cause of acute febrile illness in northeast India, can have various neurological presentations. A high index of suspicion is required to arrive at an early diagnosis and prompt management. Here, we presented an unusual case of scrub typhus presenting as bilateral sixth nerve palsy and papilledema. We conclude that in any case of acute febrile illness with sudden-onset focal neurological deficit, scrub typhus should be considered as a differential diagnosis, especially in an endemic area, as central nervous system involvement in scrub typhus has a fair outcome with good treatment.
